# Circulating concentrations of bile acids and prevalent chronic kidney disease among newly diagnosed type 2 diabetes: a cross-sectional study

**DOI:** 10.1186/s12937-024-00928-2

**Published:** 2024-03-02

**Authors:** Tingting Geng, Qi Lu, Limiao Jiang, Kunquan Guo, Kun Yang, Yun-Fei Liao, Meian He, Gang Liu, Huiru Tang, An Pan

**Affiliations:** 1 Department of Epidemiology and Biostatistics, Ministry of Education Key Laboratory of Environment and Health, School of Public Health, Tongji Medical College, Huazhong University of Science and Technology, Wuhan, Hubei Province China; 2 Department of Nutrition and Food Hygiene, School of Public Health, Institute of Nutrition, Fudan University, Shanghai, China; 3 Department of Nutrition and Food Hygiene, Hubei Key Laboratory of Food Nutrition and Safety, School of Public Health, Tongji Medical College, Huazhong University of Science and Technology, Wuhan, Hubei Province China; 4 Affiliated Dongfeng Hospital, Hubei University of Medicine, Shiyan, China; 5 Department of Endocrinology, Union Hospital, Huazhong University of Science and Technology, Wuhan, China; 6 Department of Occupational and Environmental Health, School of Public Health, Tongji Medical College, Huazhong University of Science and Technology, Wuhan, China; 7 State Key Laboratory of Genetic Engineering, School of Life Sciences, Laboratory of Metabonomics and Systems Biology, Human Phenome Institute, Zhongshan Hospital, Fudan University, Shanghai, China

**Keywords:** Bile acids, Chronic kidney disease, eGFR, Type 2 diabetes

## Abstract

**Background:**

The relationship between circulating bile acids (BAs) and kidney function among patients with type 2 diabetes is unclear. We aimed to investigate the associations of circulating concentrations of BAs, particularly individual BA subtypes, with chronic kidney disease (CKD) in patients of newly diagnosed type 2 diabetes.

**Methods:**

In this cross-sectional study, we included 1234 newly diagnosed type 2 diabetes who participated in an ongoing prospective study, the Dongfeng-Tongji cohort. Circulating primary and secondary unconjugated BAs and their taurine- or glycine-conjugates were measured using ultraperformance liquid chromatography-tandem mass spectrometry. CKD was defined as eGFR < 60 ml/min per 1.73 m^2^. Logistic regression model was used to compute odds ratio (OR) and 95% confidence interval (CI).

**Results:**

After adjusting for multiple testing, higher levels of total primary BAs (OR per standard deviation [SD] increment: 0.78; 95% CI: 0.65–0.92), cholate (OR per SD: 0.78; 95% CI: 0.66–0.92), chenodeoxycholate (OR per SD: 0.81; 95% CI: 0.69–0.96), glycocholate (OR per SD: 0.81; 95% CI: 0.68–0.96), and glycochenodeoxycholate (OR per SD: 0.82; 95% CI: 0.69–0.97) were associated with a lower likelihood of having CKD in patients with newly diagnosed type 2 diabetes. No significant relationships between secondary BAs and odds of CKD were observed.

**Conclusions:**

Our findings showed that higher concentrations of circulating unconjugated primary BAs and their glycine-conjugates, but not taurine-conjugates or secondary BAs, were associated with lower odds of having CKD in patients with type 2 diabetes.

**Supplementary information:**

The online version contains supplementary material available at 10.1186/s12937-024-00928-2.

## Introduction

Type 2 diabetes (T2D) is a global public health crisis with high morbidity and mortality [1]. The number of people living with T2D has been increasing, and it is projected to exceed 780 million by 2045 [2]. Diabetic kidney disease is one of the major microvascular complications, affecting nearly 50% of those with diabetes [3, 4]. According to data from the health care systems in the United States, the incidence of chronic kidney disease (CKD) among patients with diabetes has remained persistently high, with a total incidence of 64 cases per 1000 person-years between 2019 and 2020 [5]. Therefore, identifying interventions that can prevent or delay the decline of kidney function among individuals with diabetes is of utmost importance.

Bile acids (BAs), have been recognized as critical signalling molecules that facilitate communications between tissues, starting from liver where produced, through the intestine where modified, to the organs where they exert multiple physiological effects. These effects include facilitating intestinal lipid absorption, acting as substrates for the gut microbiome, and regulating cellular process [6, 7]. BAs also play a critical role in maintaining lipid and glucose homeostasis via activation of specific receptors, e.g., G protein-coupled bile acid receptor 1 (GPBAR1) and nuclear farnesoid X receptor (FXR) [6, 8–11]. Emerging evidence has shown circulating BAs were positively correlated with obesity (general adiposity and visceral adiposity), insulin resistance, and diabetes [12–16]. Moreover, recent findings from the POUNDS lost trial indicated that the reductions in specific subtypes of BAs in response to diet interventions were associated with improved adiposity, glucose metabolism and insulin sensitivity [17, 18]. These cardiometabolic traits are all well-known risk factors for CKD [19]. In addition, experimental studies have demonstrated that the activation of BA receptors, GPBAR1 and FXR, could protect against kidney injury by inhibiting the inflammatory pathways, reducing oxidative stress, and enhancing mitochondrial respiration [20–22]. In addition, one small retrospective study including 184 patients with diabetic kidney disease found that a higher level of total BAs was associated with a lower risk of progression to end-stage renal disease [23]. However, there is a lack of epidemiological evidence on the relationship between circulating concentrations of BAs, particularly the individual BA subtypes, and kidney function among patients with diabetes.

In this study, we comprehensively assessed the associations of circulating BAs, including subtypes of primary and secondary BAs with odds of having CKD among patients with newly diagnosed T2D using data from the Dongfeng-Tongji cohort study.

## Methods

### Study population

Our study is a subsample of the Dongfeng-Tongji cohort, which is a large and dynamic prospective cohort for long-term study of genetic and lifestyle determinants of chronic diseases of Chinese adults. Details on the rationale and design of the study have been presented previously [24]. Briefly, the Dongfeng-Tongji cohort initially enrolled 27,009 (14,957 women and 12,052 men) retired employees of the Dongfeng Motor Corporation, Shiyan City, China between 2008 and 2010, and additionally enrolled 14,120 retired workers in 2013. Socio-demographic information, anthropometric parameters, lifestyle factors (smoking status, alcohol intake, diet, physical activity, sleep), medical history, and medication history were elicited by trained interviewers. In addition, participants also provided overnight fasting blood samples.

**Fig. 1:**
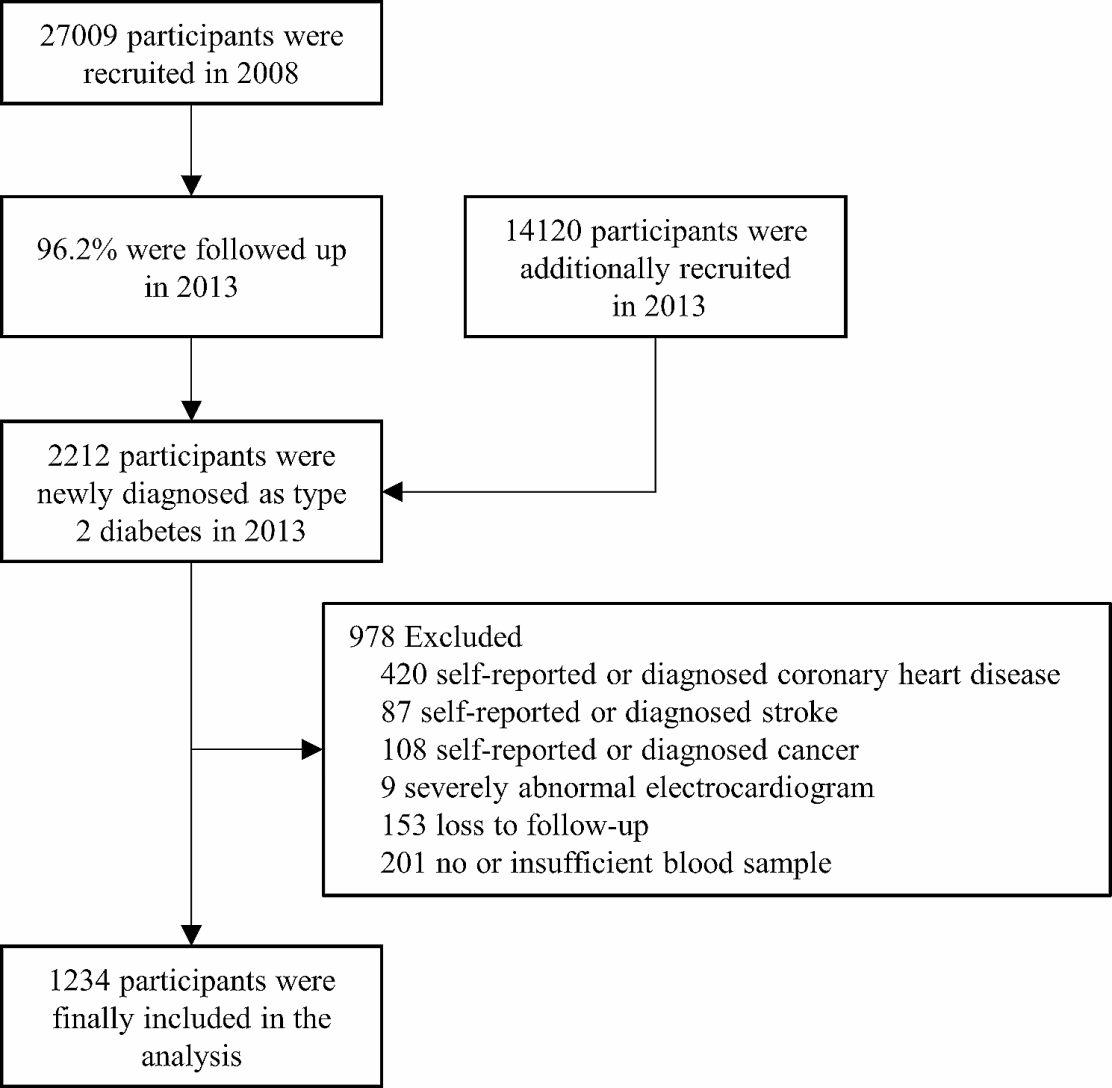
Flow chart of the study

A total of 2212 participants were newly diagnosed as T2D in 2013. New-onset T2D were defined as fasting plasma glucose (FPG) ≥ 7.0 mmol/L or haemoglobin A1c (HbA1c) ≥ 6.5%, but not on any antidiabetic medications nor being diagnosed as diabetes before the physical examination in 2013. After further exclusion of participants with coronary heart disease (*N* = 420), stroke (*N* = 87), severe abnormal electrocardiogram (*N* = 9), or cancer (*N* = 108), who were lost to follow-up (*N* = 153), and those without sufficient blood samples for BAs measurement (*N* = 201), 1234 participants were eligible for final analysis [25]. The flow chart of the study population is shown in Fig. 1.

The study was approved by the Ethics and Human Subject Committees of the Tongji Medical College, and all participants signed written informed consents.

### Measurement of subtypes of BAs

Fasting blood samples were collected from consenting participants at recruitment, separated by components, and stored at -80 °C until measurement. After solvent extraction, plasma samples were detected for BAs in the multiple reaction monitoring mode on a ultraperformance liquid chromatography, coupled to tandem mass spectrometry (UPLC-MS/MS, Agilent Technologies, USA), according to the previously optimized method with minor modifications [26]. The target BAs included unconjugated primary BAs (cholate [CA] and chenodeoxycholate [CDCA]) and their amino acid conjugates (glycocholate [GCA], taurocholate [TCA], glycochenodeoxycholate [GCDCA], taurochenodeoxycholate [TCDCA]), as well as unconjugated secondary BAs (deoxycholate [DCA], lithocholate [LCA], hyocholic acid [HCA], and ursodeoxycholate [UDCA]) and conjugated secondary BAs (glycodeoxycholate [GDCA], taurodeoxycholate [TDCA], glycolithocholate [GLCA], taurolithocholic acid [TLCA], glycohyocholic acid [GHCA], taurohyocholic acid [THCA], glycoursodeoxycholate [GUDCA] and tauroursodeoxycholic acid [TUDCA]). BAs were quantified by calibration curves constructed from standards and corresponding isotopically labelled internal standards using MassHunter Workstation software (Agilent, Version B.08.00). BAs with detection rates below 80% (except for LCA) were omitted from subsequent analyses. The values below the limits of detection were imputed with the half minimum across all subjects. The intra-assay CVs ranged from 3.6 to 18.6% for all included BAs.

### Assessment of the covariates

At recruitment, the well-trained interviewers conducted face-to-face interviews with participants using a structured questionnaire and collected information on various covariates. These included age, sex, education, tobacco use, alcohol intake, physical activity and medical history. For cigarette smoking and alcohol drinking, participants were categorized as never, former and current smokers/drinkers. Participants were defined as current smokers/drinkers if they had smoked at least one cigarette a day for more than six month/had consumed alcohol at least once per week for six months or longer. Body mass index (BMI, kg/m^2^) was calculated via body weight in kilograms divided by square of height in meters. Regular physical activity was defined as exercise for at least 20 min per week for over half a year. Pre-existing hypertension was defined as meeting any of the following criteria: (1) self-reported physician-diagnosed cases; (2) undertaking the anti-hypertensive medications; (3) blood pressure measurements of 140/90 mm Hg or higher. In addition, plasma levels of FPG, HbA1c, triglycerides (TG), total cholesterol (TC), low-density lipoprotein cholesterol (LDL-C), and high-density lipoprotein cholesterol (HDL-C) were determined at the central laboratory of the Dongfeng Hospital following standard laboratory procedures.

### Assessment of the outcomes

We evaluated two outcomes: the prevalent CKD and baseline estimated glomerular filtration rate (eGFR). Serum creatinine was determined using the sarcosine oxidase assay method by the ArchitectCi8200 automatic analyzer (ABBOTT Laboratories. Abbott Park, Illinois, USA), and the baseline eGFR was calculated using the Chronic Kidney Disease Epidemiology Collaboration equation [27]. The equation expressed as a single equation is eGFR = 141 × min(Scr/ κ, 1)^α^ × max(Scr/ κ, 1)^−1.209^ × 0.993^Age^×1.018[if female], where Scr is serum creatinine, κ is 0.7 for females and 0.9 for males, α is -0.329 for females and − 0.411 for males, min indicates the minimum of Scr/κ or 1, and max indicates the maximum of Scr/κ or 1. In the current study, CKD was defined as eGFR < 60 ml/min per 1.73 m^2^.

### Statistical analysis

Baseline characteristics were presented as mean (standard deviation [SD]) or median (interquartile range [IQR]) for continuous variables, and n (%) for categorical variables. Differences between participants with and without CKD status were examined using Student’s *t* test, Mann-Whitney U test, and *chi*-squared test where appropriate. Data on individual subtypes of BAs were log-transformed and z-scored before analysis.

Logistic regression models were used to compute the odds ratios (ORs) and 95% confidence intervals (95% CIs) for the associations of individual BAs with odds of having CKD. In the multivariable-adjusted model, we adjusted for age (continuous, years), sex (men, women), education level (less than high school, high school, or college and above), BMI (continuous, kg/m^2^), smoking status (never, former, or current), alcohol intake (never, former, or current), physically active (yes or no), history of hypertension (yes or no), lipid-lowering medications (yes or no), fasting glucose (continuous, mmol/L), and plasma concentrations of triglycerides (continuous, mmol/L), LDL-C (continuous, mmol/L) and HDL-C (continuous, mmol/L). The association between BAs and eGFR was examined using the generalized linear regression model with the adjustment for potential confounders abovementioned.

We stratified the analyses by age (< 65, ≥ 65 years), sex (men, women), BMI (< 24, ≥ 24 kg/m^2^), smoking status (never, ever smoking), LDL-C (< 3.3, ≥ 3.3 mmol/L), HDL-C (≤ 1.5, > 1.5 mmol/L), and TG (< 1.7, ≥ 1.7 mmol/L). The interactions of circulating BAs and the stratified factors on the odds of having CKD were tested using the likelihood ratio test by including the multiplicative terms in the multivariable-adjusted models. The relationships between concentrations of BAs and the odds of CKD were also evaluated on a continuous scale using restricted cubic spline analysis. We performed several sensitivity analyses to test the robustness of our findings. First, as liver function may affect the observed associations, we further adjusted for the liver function biomarkers including alanine aminotransferase [ALT] and aspartate aminotransferase [AST]. Second, we additionally adjusted for a diet score based on intakes of vegetables, fruits, and meats [28]. Finally, we also repeated the analyses where cases of CKD were redefined as having an eGFR of less than 60 ml/min per 1.73 m², or proteinuria levels of 1 + or higher.

All analyses were performed in Stata statistical software, release 15.1 (StataCorp LP, College Station, Texas). The false discovery rate (FDR) was calculated using the B-H method to account for multiple testing, and *P* value < 0.05 after B-H FDR adjustment was considered as statistically significant.

## Results

Distributions of baseline characteristics of the study population on the basis of CKD status are shown in Table 1. Of the 1234 participants (mean age, 63.7 years [SD, 7.9]) in the study, 662 (53.7%) were women and 193 (15.6%) had CKD. Prevalent CKD patients were older, and more likely to be never smokers, had higher levels of TC, TG, and lower levels of LDL-C, HDL-C, ALT, and diastolic blood pressure compared with those without CKD. A total of 14 BAs were included in the final analysis, with THCA, TUDCA, GLCA, and TLCA being excluded due to a low detection rate (≤ 60%).

**Table 1:** Baseline characteristics of study population

	Total population	eGFR, ml/min/1.73m^2^		P*
		< 60	≥ 60	
N	1234	193	1041	
Age, years	63.7 (7.9)	66.8 (8.2)	63.1 (7.7)	< 0.001
Sex				0.10
Men	572 (46.4)	79 (40.9)	493 (47.4)	
Women	662 (53.7)	114 (59.1)	548 (52.6)	
Education attainment				0.21
Less than high school	701 (56.8)	109 (56.5)	592 (56.9)	
High school or equivalent	402 (32.6)	57 (29.5)	345 (33.1)	
College or above	131 (10.6)	27 (14.0)	104 (10.0)	
Smoking status				< 0.001
Never	897 (72.7)	157 (81.4)	740 (71.1)	
Current	216 (17.5)	14 (7.3)	202 (19.4)	
Former	121 (9.8)	22 (11.4)	99 (9.5)	
Drinking status				0.07
Never	836 (67.8)	144 (74.6)	692 (66.5)	
Current	352 (28.5)	42 (21.8)	310 (29.8)	
Former	46 (3.7)	7 (3.6)	39 (3.8)	
Regular physical activity	1090 (88.3)	173 (89.6)	917 (88.1)	0.54
Hypertension at baseline	855 (69.3)	132 (68.4)	723 (69.5)	0.77
Clinical parameters				
Waist to hip ratio	0.90 (0.06)	0.90 (0.06)	0.90 (0.06)	0.68
Waist circumference, cm	87.2 (9.3)	87.3 (9.9)	87.1 (9.2)	0.80
Hip circumference, cm	97.1 (7.2)	97.4 (7.7)	97.0 (7.1)	0.49
Body mass index, kg/m^2^	25.5 (3.6)	25.6 (4.0)	25.5 (3.5)	0.85
Total cholesterol, mmol/L	5.08 (1.13)	5.25 (1.18)	5.05 (1.12)	0.02
Triglycerides, mmol/L	1.91 (1.76)	2.53 (3.50)	1.79 (1.15)	< 0.001
LDL-C, mmol/L	2.86 (0.90)	2.73 (0.83)	2.88 (0.91)	0.03
HDL-C, mmol/L	1.43 (0.41)	1.37 (0.40)	1.44 (0.41)	0.01
Systolic blood pressure, mmHg	144.7 (23.2)	144.0 (22.4)	144.9 (23.3)	0.62
Diastolic blood pressure, mmHg	82.9 (13.0)	81.1 (13.4)	83.3 (12.8)	0.04
Haemoglobin A1c, %	6.7 (1.6)	6.5 (1.2)	6.8 (1.6)	0.14
Fasting plasma glucose, mmol/L	8.2 (2.3)	8.2 (2.3)	8.1 (2.3)	0.54
γ-glutamyl transferase, U/L	37.5 (55.2)	35.8 (57.9)	37.8 (54.7)	0.64
Alkaline phosphatase, U/L	92.9 (28.8)	91.6 (26.7)	93.2 (29.1)	0.47
Total bilirubin, µmol/L	14.3 (5.8)	13.7 (6.1)	14.4 (5.7)	0.15
Alanine aminotransferase, U/L	26.4 (23.1)	22.3 (12.7)	27.2 (24.4)	0.006
Aspartate aminotransferase, U/L	26.7 (16.7)	25.3 (13.6)	27.0 (17.2)	0.19

Data are presented as mean (SD) for continuous variable or n (%) for categorical variables. *Differences between groups were compared using the Chi-square test for categorical variables, the Student’s t-test for normally distributed continuous variables, and the Wilcoxon rank sum test for non-normally distributed continuous variables.

Abbreviation: eGFR, estimated glomerular filtration rate; LDL-C, low-density lipoprotein cholesterol; HDL-C, high-density lipoprotein cholesterol

**Fig. 2:**
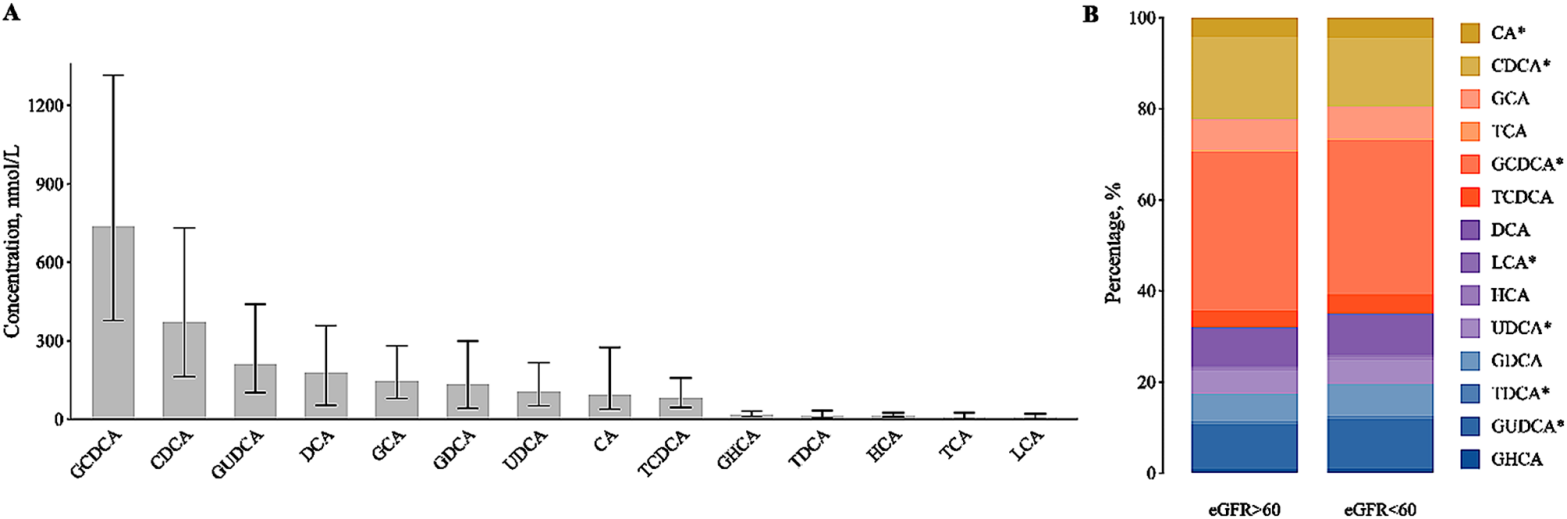
Distribution of bile acids **A**: Results are shown as bars denoting the medians and error bars denoting the interquartile ranges. **B**: Differences in the composition of bile acids in participants with eGFR ≥ 60 ml/min/1.73m^2^ and eGFR < 60 ml/min/1.73m^2^. *P < 0.05. Abbreviation: eGFR, estimated glomerular filtration rate

The distribution of BAs is shown in Fig. 2A, where GCDCA had the highest concentration (median: 736.1 nmol/L), followed by CDCA and GUDCA. Compared with participants without CKD, those with CKD had lower percentages of CA, CDCA, GCDCA, LCA and UDCA, and higher percentages of TDCA and GUDCA (Fig. 2B).

In the multivariate-adjusted model, we observed that total primary BAs, unconjugated primary BAs (CA and CDCA), and their glycine-conjugates (GCA and GCDCA) were significantly associated with lower odds of CKD among patients with newly diagnosed T2D (P_FDR_ < 0.05). RCS analysis showed a linear relationship between primary BAs and CKD (Supplemental Fig. 1). The OR per SD increment (95% CI) for CKD was 0.78 (0.65–0.92) for total primary BAs, 0.78 (0.66–0.92) for CA, 0.81 (0.69–0.96) for CDCA, 0.81 (0.68–0.96) for GCA, and 0.82 (0.69–0.97) for GCDCA (Table 2). In addition, higher levels of total primary BAs (β per SD increment: 2.09; 95% CI: 0.63–3.56), CA (β per SD increment: 2.75; 95% CI: 1.30–4.20) and CDCA (β per SD increment: 2.52; 95% CI: 1.06–3.97) were associated with higher levels of eGFR at baseline among patients with newly diagnosed T2D, while the association with GCA and GCDCA were not statistically significant (Table 2). There was no significant relationship between secondary BAs (unconjugated and conjugated subtypes) and odds of CKD or eGFR at baseline in patients with new-onset T2D (Table 3).

**Table 2:** Association of primary BAs with CKD and eGFR among newly-diagnosed type 2 diabetes

	CKD			eGFR	
	OR (95% CI)*	P FDR		β (95% CI)*	P FDR
Total primary BAs	0.78 (0.65, 0.92)	0.01		2.09 (0.63, 3.56)	0.01
Unconjugated primary BAs					
CA	0.78 (0.66, 0.92)	0.01		2.75 (1.30, 4.20)	0.001
CDCA	0.81 (0.69, 0.96)	0.03		2.52 (1.06, 3.97)	0.003
Conjugated primary BAs					
GCA	0.81 (0.68, 0.96)	0.03		0.87 (-0.61, 2.34)	0.35
TCA	0.85 (0.72, 1.00)	0.05		0.77 (-0.69, 2.23)	0.35
GCDCA	0.82 (0.69, 0.97)	0.03		0.97 (-0.51, 2.44)	0.35
TCDCA	0.91 (0.77, 1.08)	0.27		-0.16 (-1.62, 1.31)	0.84

*Models were adjusted for age, sex, education, body mass index, smoking status, alcohol consumption, physical activity, hypertension, use of lipid-lowering medication, fasting plasma glycose, triglycerides, low-density lipoprotein cholesterol and high-density lipoprotein cholesterol

Abbreviation: BA, bile acid CKD; chronic kidney disease; eGFR, estimated glomerular filtration rate; OR, odd ratio; FDR, false discovery rate

**Table 3:** Association of secondary BAs with CKD and eGFR among newly-diagnosed type 2 diabetes

	CKD			eGFR	
	OR (95% CI)*	P FDR		β (95% CI)*	P FDR
Total secondary BAs	0.88 (0.74, 1.04)	0.75		2.05 (0.58, 3.53)	0.05
Unconjugated secondary BAs					
DCA	0.92 (0.79, 1.08)	0.75		1.19 (-0.27, 2.65)	0.20
LCA	1.00 (0.85, 1.18)	0.99		1.54 (0.06, 3.03)	0.09
HCA	0.92 (0.78, 1.08)	0.75		1.90 (0.44, 3.36)	0.05
UDCA	0.94 (0.81, 1.11)	0.82		1.60 (0.14, 3.05)	0.09
Conjugated secondary BAs					
GDCA	0.92 (0.78, 1.08)	0.75		0.83 (-0.63, 2.28)	0.34
TDCA	0.97 (0.82, 1.14)	0.94		-0.27 (-1.72, 1.19)	0.75
GUDCA	0.95 (0.81, 1.12)	0.82		0.85 (-0.61, 2.31)	0.34
GHCA	1.00 (0.85, 1.18)	0.99		0.24 (-1.25, 1.73)	0.75

*Models were adjusted for age, sex, education, body mass index, smoking status, alcohol consumption, physical activity, hypertension, use of lipid-lowering medication, fasting plasma glycose, triglycerides, low-density lipoprotein cholesterol and high-density lipoprotein cholesterol.

Abbreviation: BA, bile acid CKD; CKD, chronic kidney disease; eGFR, estimated glomerular filtration rate; OR, odd ratio; FDR, false discovery rate

**Fig. 3:**
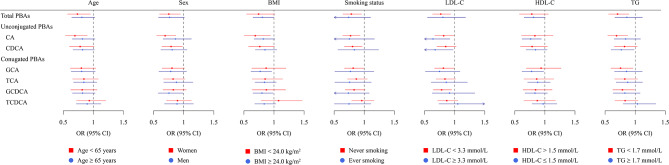
Stratified analyses between primary BAs and odds of CKD Models were adjusted for age, sex, education, BMI, smoking status, alcohol consumption, physical activity, hypertension, use of lipid-lowering medication, fasting plasma glycose, TG, LDL-C and HDL-C. Abbreviation: BA, bile acid; BMI, body mass index; LDL-C, low-density lipoprotein cholesterol; HDL-C, high-density lipoprotein cholesterol; TG, triglycerides; OR, odds ratio; CKD, chronic kidney disease

Subgroup analyses showed consistent results when stratified by age, sex, BMI and lipids. No significant risk heterogeneity between BAs and stratified factors in relation to odds of having CKD among patients with newly diagnosed T2D was observed (*P*_− interaction_>0.05 for all; Fig. 3). In the sensitivity analyses, with the additional adjustment for ALT and AST, the results were materially unchanged, except for GCA and GCDCA, where *P* values became borderline significant (both *P*_FDR_ = 0.06) (Supplemental Table 1). Second, the results were almost unchanged with the adjustment for the diet score (Supplemental Table 2). Finally, the associations between BAs and CKD remained largely consistent, though the strength of associations was slightly attenuated. Specifically, the OR per SD increment for CKD was 0.82 (95% CI: 0.71–0.96) for total primary BAs, 0.82 (0.71–0.96) for CA, 0.85 (0.73–0.98) for CDCA, 0.82 (0.70–0.95) for GCA, and 0.85 (0.73, 0.99) for GCDCA (Supplemental Table 3).

## Discussion

To our best knowledge, this is the first investigation that examined the associations between the profile of BAs and likelihood of having CKD among patients with new-onset T2D. Our data showed that a higher level of certain primary BAs, namely CA, CDCA, GCA and GCDCA were associated with a lower likelihood of having CKD among new-onset T2D. The associations were consistent across subgroup analyses. However, we did not observe significant associations between secondary BAs and odds of having CKD.

Recently, BAs have gained increasing attention as important signalling molecules in metabolic disease and traits, e.g., obesity [12, 13], diabetes [15, 16], non-alcoholic fatty liver disease [29, 30], and cardiovascular disease [31–33]; however, the relationship between circulating BAs profile and kidney function is limited. A retrospective cohort study including 184 patients with biopsy-proven diabetic kidney disease showed that lower levels of total BAs were associated with a higher risk of developing end-stage kidney disease [23]. Our results expanded the knowledge to this domain by demonstrating that the relationship between total BAs and kidney function decline among patients with diabetes was mainly driven by primary BAs.

In addition, the Chronic Renal Insufficiency Cohort (CRIC) study including 3,147 patients with CKD found that higher levels of DCA, a type of secondary BA, were associated with a higher risk of progression to end-stage kidney disease and all-cause mortality [34]. However, in our study, we did not find any significant relationship between secondary BAs and odds of having CKD or eGFR at baseline among patients with newly diagnosed T2D.

To our best knowledge, only one cross-sectional study has systematically investigated the relationship between BA profile and CKD. In this study, Wang et al. found that conjugated primary BAs (GCDCA-3-glucu), and conjugated secondary BAs (TCA, TDCA, TUDCA) were significantly different in 223 participants of end-stage kidney disease and 69 individuals without end-stage kidney disease [35]. In contrast, our study of patients with new-onset T2D showed that, besides the glycine-conjugated primary BAs, unconjugated primary BAs (CA, and CDCA) were also significantly associated with the odds of having CKD, but not the secondary BAs. The differences might be explained by several possibilities. First, our study focused on newly diagnosed T2D whereas the controls in Wang et al’s study were healthy individuals. Further, the outcomes were CKD defined as eGFR < 60 ml/min per 1.73 m^2^ in our analysis while Wang et al’s study focused on end-stage kidney disease (hemodialysis patients). In addition, both analyses were limited to the cross-sectional study design and relatively small sample size; future studies with prospective data and larger sample sizes are needed to better understand the relationship between the BAs profile and kidney function decline.

Although the exact underlying mechanism linking the BA profile and CKD is not fully understood, the putative relationship might be explained through both direct and indirect pathways. BA receptors, FXR and GPBAR1 may be involved in protecting kidney function in diabetes and obesity directly. In experimental studies, activation of GPBAR1and FXR resulted in decreased inflammation, renal oxidative stress, and lipid accumulation in mice with diabetes and obesity [20, 36]. Further, the expression of renal FXR and GPBAR1 can induce effects similar to caloric restriction, thereby reversing age-related kidney injuries, e.g., mitochondrial dysfunction, podocyte injury, and fibronectin accumulation [21]. However, future studies are required to elucidate the precise mechanisms involved.

Our research on the relationship between circulating BAs and the prevalence of CKD in newly diagnosed T2D patients highlights several potential clinical applications. These include using specific BAs as biomarkers for early CKD detection in T2D patients, informing therapeutic strategies by exploring treatments that modulate levels of BAs, and emphasizing the need for regular monitoring of kidney function and BAs for preventative measures. This understanding could guide future longitudinal studies to investigate prospective relationships and mechanisms, enhancing our knowledge of how bile acids influence CKD progression in patients with T2D.

The strengths of our study include a comprehensive assessment of subtypes of BAs and relatively large sample size. Furthermore, we conducted the study among patients with newly new-onset T2D, which could minimize the possiblity of the effect of medications for diabetes on the observed associations. Several limitations should be acknowledged to aid in the interpretation of our results. First, the current analysis was based on cross-sectional data, thus the temporal relations cannot be determined, and the relationship between BAs profile and CKD might be bidirectional. Second, CKD was defined based only on eGFR as quantitative data on albuminuria was not available, which may lead to misclassification. However, the results were consistent when using eGFR and dipstick proteinuria to define CKD in the sensitivity analysis. Future research should aim to include both eGFR and Urine Albumin-to-Creatinine Ratio measures to provide a more complete understanding of kidney function and disease progression, especially in diabetic populations. In addition, chronicity data for eGFR were not available similar as many other epidemiological studies with large sample size; hence, we cannot completely rule out the possibility of acute kidney disease cases in the prevalent CKD. Third, no information on end-stage kidney disease was available, which limited our capacity to explore the associations between BAs and kidney function at more severe stages of CKD. Future studies are warranted to evaluate the role of BAs across different stages of CKD, especially within the context of diabetes. Fourth, our findings were based on middle-aged and older Chinese who were patients with newly diagnosed T2D but without comorbid conditions like heart disease, stroke, abnormal electrocardiograms, or cancer, which may limit the generalizability of our findings to other populations. Fifth, although our study population were restricted to new-onset T2D before treatment, other factors such as data on use of antibiotics or pre-biotics, and gut microbiota may also affect the metabolism of BAs. Sixth, we were not able to examine the associations of fecal BAs with CKD among patients with diabetes due to a lack of the information. Finally, as in any observational study, residual confounding cannot be completely ruled out.

## Conclusion

We found that among patients with newly diagnosed T2D, those with lower levels of circulating unconjugated primary BAs and their glycine-conjugates had a high likelihood of having CKD. However, taurine-conjugated primary BAs or secondary BAs were not associated with CKD. Future prospective studies are warranted to corroborate our results. Our findings, if confirmed, may provide evidence on novel intervention targets for CKD prevention in patients with T2D.

### Electronic supplementary material

Below is the link to the electronic supplementary material.

Supplementary Material 1

## Data Availability

The datasets used and/or analysed during the current study available from the corresponding author on reasonable request.
