# Supplementary material for: Circulating concentrations of bile acids and prevalent chronic kidney disease among newly diagnosed type 2 diabetes: a cross-sectional study
Source: Nutr J. 2024 Mar 2;23:28. doi: 10.1186/s12937-024-00928-2 (PMC10908139; doi:10.1186/s12937-024-00928-2)
Supplement: Supplementary file 1 — Supplementary Material 1 [file 12937_2024_928_MOESM1_ESM.docx]

**Supplemental Material**

**Circulating concentrations of bile acids and prevalent chronic kidney disease among newly diagnosed type 2 diabetes: a cross-sectional study**

| **Supplemental Table 1.** Association of BAs with CKD and eGFR among newly-diagnosed type 2 diabetes after further adjustment for ALT and AST | | | | | |
| --- | --- | --- | --- | --- | --- |
|  | CKD | |  | eGFR | |
|  | OR (95% CI)* | *P* FDR |  | β (95% CI)* | *P* FDR |
| Total primary BAs | 0.79 (0.66, 0.94) | 0.03 |  | 2.26 (0.76, 3.76) | 0.007 |
| Unconjugated primary BAs | |  |  |  |  |
| CA | 0.79 (0.66, 0.93) | 0.03 |  | 2.75 (1.30, 4.20) | 0.001 |
| CDCA | 0.82 (0.69, 0.96) | 0.04 |  | 2.53 (1.07, 3.98) | 0.002 |
| Conjugated primary BAs | |  |  |  |  |
| GCA | 0.83 (0.69, 0.99) | 0.06 |  | 0.98 (-0.55, 2.51) | 0.29 |
| TCA | 0.87 (0.74, 1.04) | 0.14 |  | 0.88 (-0.65, 2.40) | 0.30 |
| GCDCA | 0.83 (0.70, 0.99) | 0.06 |  | 1.09 (-0.43, 2.60) | 0.28 |
| TCDCA | 0.94 (0.79, 1.11) | 0.45 |  | -0.09 (-1.61, 1.44) | 0.91 |
|  | | | | | |
| Total secondary BAs | 0.89 (0.75, 1.05) | 0.87 |  | 2.07 (0.60, 3.55) | 0.05 |
| Unconjugated secondary BAs | |  |  |  |  |
| DCA | 0.93 (0.79, 1.09) | 0.87 |  | 1.13 (-0.34, 2.60) | 0.24 |
| LCA | 1.00 (0.84, 1.18) | 1.00 |  | 1.51 (0.03, 3.00) | 0.10 |
| HCA | 0.92 (0.78, 1.09) | 0.87 |  | 1.90 (0.43, 3.36) | 0.05 |
| UDCA | 0.95 (0.81, 1.11) | 0.92 |  | 1.59 (0.13, 3.05) | 0.10 |
| Conjugated secondary BAs | |  |  |  |  |
| GDCA | 0.93 (0.79, 1.09) | 0.87 |  | 0.78 (-0.67, 2.24) | 0.37 |
| TDCA | 0.99 (0.84, 1.17) | 1.00 |  | -0.30 (-1.76, 1.15) | 0.75 |
| GUDCA | 0.96 (0.81, 1.13) | 0.95 |  | 0.91 (-0.56, 2.38) | 0.34 |
| GHCA | 1.03 (0.87, 1.22) | 0.96 |  | 0.24 (-1.28, 1.77) | 0.75 |
| *Models were adjusted for age, sex, education, body mass index, smoking status, alcohol consumption, physical activity, hypertension, use of lipid-lowering medication, fasting plasma glycose, triglycerides, low-density lipoprotein cholesterol, high-density lipoprotein cholesterol, ALT, and AST.  Abbreviation: BA, bile acid; CKD, chronic kidney disease; eGFR, estimated glomerular filtration; FDR, false discovery rate. ALT, alanine aminotransferase; AST, aspartate aminotransferase. | | | | | |

| **Supplemental Table 2.** Association of BAs with CKD and eGFR among newly-diagnosed type 2 diabetes after further adjustment for diet score | | | | | |
| --- | --- | --- | --- | --- | --- |
|  | CKD | |  | eGFR | |
|  | OR (95% CI)* | *P* FDR |  | β (95% CI)* | *P* FDR |
| Total primary BAs | 0.78 (0.66, 0.93) | 0.02 |  | 2.06 (0.60, 3.53) | 0.01 |
| Unconjugated primary BAs | |  |  |  |  |
| CA | 0.78 (0.66, 0.93) | 0.02 |  | 2.70 (1.25, 4.15) | 0.002 |
| CDCA | 0.82 (0.69, 0.96) | 0.03 |  | 2.46 (1.00, 3.92) | 0.003 |
| Conjugated primary BAs | |  |  |  |  |
| GCA | 0.81 (0.68, 0.96) | 0.03 |  | 0.87 (-0.61, 2.34) | 0.35 |
| TCA | 0.85 (0.72, 1.00) | 0.06 |  | 0.75 (-0.72, 2.21) | 0.37 |
| GCDCA | 0.82 (0.69, 0.97) | 0.03 |  | 0.94 (-0.53, 2.42) | 0.35 |
| TCDCA | 0.91 (0.77, 1.08) | 0.29 |  | -0.19 (-1.66, 1.27) | 0.80 |
|  | | | | | |
| Total secondary BAs | 0.88 (0.75, 1.04) | 0.76 |  | 2.01 (0.54, 3.48) | 0.06 |
| Unconjugated secondary BAs | |  |  |  |  |
| DCA | 0.92 (0.78, 1.08) | 0.76 |  | 1.23 (-0.23, 2.69) | 0.18 |
| LCA | 1.00 (0.84, 1.18) | 0.97 |  | 1.57 (0.09, 3.05) | 0.09 |
| HCA | 0.92 (0.78, 1.09) | 0.76 |  | 1.85 (0.39, 3.31) | 0.06 |
| UDCA | 0.95 (0.81, 1.12) | 0.90 |  | 1.52 (0.06, 2.98) | 0.09 |
| Conjugated secondary BAs | |  |  |  |  |
| GDCA | 0.91 (0.78, 1.07) | 0.76 |  | 0.87 (-0.58, 2.33) | 0.36 |
| TDCA | 0.97 (0.82, 1.14) | 0.90 |  | -0.24 (-1.69, 1.22) | 0.78 |
| GUDCA | 0.96 (0.81, 1.13) | 0.90 |  | 0.78 (-0.69, 2.24) | 0.38 |
| GHCA | 1.01 (0.85, 1.19) | 0.97 |  | 0.22 (-1.27, 1.71) | 0.78 |
| *Models were adjusted for age, sex, education, body mass index, smoking status, alcohol consumption, physical activity, hypertension, use of lipid-lowering medication, fasting plasma glycose, triglycerides, low-density lipoprotein cholesterol, high-density lipoprotein cholesterol, and diet score.  Abbreviation: BA, bile acid; CKD, chronic kidney disease; eGFR, estimated glomerular filtration; FDR, false discovery rate. ALT, alanine aminotransferase; AST, aspartate aminotransferase. | | | | | |

**Supplemental table 3.** Association between BAs and CKD among newly-diagnosed type 2 diabetes

|  | CKD^a^ | |
| --- | --- | --- |
|  | OR (95% CI)^b^ | *P*_FDR_ |
| Total primary BAs | 0.82 (0.71, 0.96) | 0.03 |
| Unconjugated primary BAs |  |  |
| CA | 0.82 (0.71, 0.96) | 0.03 |
| CDCA | 0.85 (0.73, 0.98) | 0.046 |
| Conjugated primary BAs |  |  |
| GCA | 0.82 (0.70, 0.95) | 0.03 |
| TCA | 0.87 (0.74, 1.00) | 0.07 |
| GCDCA | 0.85 (0.73, 0.99) | 0.046 |
| TCDCA | 0.96 (0.82, 1.11) | 0.56 |
|  |  |  |
| Total secondary BAs | 0.86 (0.73, 1.00) | 0.39 |
| Unconjugated secondary BAs |  |  |
| DCA | 0.94 (0.81, 1.09) | 0.74 |
| LCA | 0.99 (0.85, 1.15) | 0.88 |
| HCA | 0.96 (0.82, 1.12) | 0.86 |
| UDCA | 0.92 (0.79, 1.06) | 0.74 |
| Conjugated secondary BAs |  |  |
| GDCA | 0.94 (0.81, 1.09) | 0.74 |
| TDCA | 0.98 (0.84, 1.13) | 0.86 |
| GUDCA | 0.93 (0.80, 1.08) | 0.74 |
| GHCA | 0.98 (0.84, 1.14) | 0.86 |

^a^CKD was defined as eGFR<60 ml/min per 1.73 m^2^ or proteinuria ≥1+.

^b^Models were adjusted for age, sex, education, body mass index, smoking status, alcohol consumption, physical activity, hypertension, use of lipid-lowering medication, fasting plasma glucose, triglycerides, low-density lipoprotein cholesterol, and high-density lipoprotein cholesterol.

Abbreviation: BA, bile acid CKD; chronic kidney disease; eGFR, estimated glomerular filtration rate; OR, odd ratio; FDR, false discovery rate.


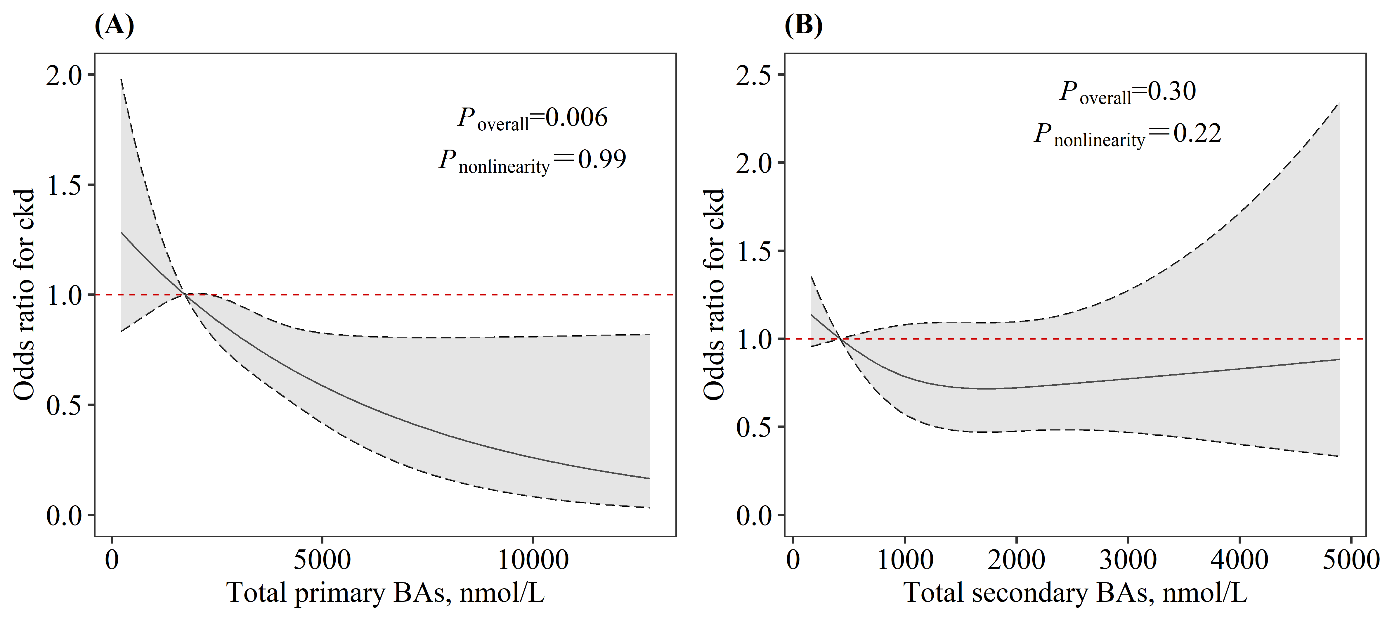


**Supplemental Figure 1.** Dose-response relationships between BAs and the odds of having CKD

Models were adjusted for age, sex, education, body mass index, smoking status, alcohol consumption, physical activity, hypertension, use of lipid-lowering medication, fasting plasma glucose, triglycerides, low-density lipoprotein cholesterol, and high-density lipoprotein cholesterol.
